# Harbour Porpoises Are Flexible Predators Displaying Context‐Dependent Foraging Behaviours

**DOI:** 10.1002/ece3.70671

**Published:** 2024-12-04

**Authors:** Johanna Stedt, Héloïse Hamel, Sara Torres Ortiz, Jakob Højer Kristensen, Magnus Wahlberg

**Affiliations:** ^1^ Department of Biology Lund University Lund Sweden; ^2^ Department of Biology University of Southern Denmark Odense Denmark; ^3^ BioNaut Odense Denmark

**Keywords:** drone, hunting behaviour, odontocetes, *Phocoena phocoena*, UAV

## Abstract

Opportunistic mobile predators can adapt their behaviour to specific foraging scenarios, allowing them to target diverse prey resources. An interesting example is the harbour porpoise ( *Phocoena phocoena* ), a marine mammal with a huge energy demand feeding on a large variety of fish, squid and shrimps. Little is known about the foraging behaviour of harbour porpoises, as observations of wild specimens are notoriously difficult to obtain. In this study, foraging was identified in almost 60% of videos from UAV recordings in Danish coastal waters during daylight hours. Observations reveal them to be flexible predators, foraging on both single fish and schools of fish, as well as individually and in groups of varying sizes. We argue that some of the observed behavioural adaptations and context‐dependent strategies for prey capture are based on information transfer and social learning. Our results provide unprecedented insights into the foraging behaviour of an opportunistic mammalian predator. Furthermore, this study highlights the importance of porpoises having access to coastal areas for energy acquisition, where they are in conflict with anthropogenic disturbances such as fisheries with the risk of bycatch.

## Graphical abstract

This study provides the first record of previously undescribed foraging behaviours of harbour porpoises. Our results indicate use of context‐dependent foraging strategies, suggesting that porpoises are flexible predators that use conditional foraging strategies and adapt their behaviour in response to environmental characteristics.

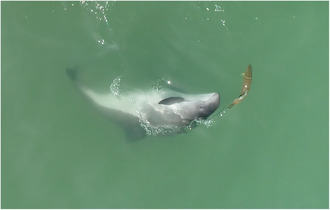

## 1 Introduction

In ecological studies, understanding animal foraging behaviour is fundamental since it directly relates to the fitness of individuals and species (Waite and Field 2007; Hintz and Lonzarich 2018) and provides information on interactions between an animal and its environment (Ydenberg, Brown, and Stephens 2007). As foraging typically requires significant energy investments, animals have strong incentives to develop foraging strategies that maximise their net energy gain (Schoener 1971). The resulting large behavioural variation in foraging behaviour can be observed both within and between species. Animals differ in the variety of food they consume, in time and effort they spend searching for and handling food, in their foraging tactics, as well as in their group size and structure (Cohen 1993; Overington, Dubois, and Lefebvre 2008; Ceia and Ramos 2015; McHuron et al. 2018).

Intraspecific behavioural variation is expected when natural selection favours individuals that are capable of expressing flexibility in their response to the environment (Alcock 2009). There is growing evidence that the degree of intraspecific behavioural variability can be used to study a species' ability to cope with changes in the environment, with increasing flexibility and behavioural plasticity providing greater resilience to habitat changes (Ameca, Chamart, and Garber 2023; Shultz et al. 2005; Charrette, Cleary, and Mooers 2006). Behaviours can thus be valuable indicators for conservation, as they represent both the current and future prospects of a species. A shift in, for example, foraging strategies could be used as a first indicator of potential changes in population size (Stephens, Brown, and Ydenberg 2007). Measuring behavioural flexibility in the animals' natural environment is, however, difficult (Hertel et al. 2020), as observations must be made at the right place and at the right time. Also, observations must not interfere with the animals' natural behaviour.

One interesting example of a predator that forages on a variety of prey species and switches diet in response to prey abundance is the harbour porpoise ( *Phocoena phocoena* ) (Ransijn et al. 2021). Harbor porpoises are small‐toothed whales living year‐round in temperate and Arctic waters (Read 1999; Carwardine 2020). They have extremely high energy demands (Rojano‐Doñate et al. 2018) and feed on a large variety of prey, ranging from schooling and solitary fish to invertebrates such as squid and shrimp (Andreasen et al. 2017; Mahfouz et al. 2017; Santos and Pierce 2003). Harbor porpoises are notoriously difficult to study in the field due to their small size, anonymous appearance and fast movements (Amundin and Amundin 1974; Elliser, Van Der Linde, and Maciver 2022). At present, little is known about the foraging strategies of harbour porpoises and their capability of behavioural adjustments to foraging contexts. Diet studies demonstrate that they predate on a range of different prey species which occupy a diversity of habitats and express a variety of predator avoidance strategies (Linehan, Gregory, and Schneider 2001; Pitcher and Wyche 1983; Turesson, Satta, and Domenici 2009). In order to maximise their net energy gain, harbour porpoises are expected to be capable of adapting their foraging strategy and behaviour to specific foraging scenarios and prey targets. However, to date, no detailed visual observations or descriptions of harbour porpoise foraging behaviours exist, except for one study which suggests that porpoises might exploit cognitive and social abilities during group hunting (Torres Ortiz et al. 2021).

Detailed studies of marine mammal foraging behaviours are challenging (Nowacek 2002), but in recent years, commercial unmanned aerial vehicles (UAVs) or drones have been increasingly used to record behavioural sequences of wild animals (Schofield et al. 2019, reviewed in Fiori et al. 2017; Raoult et al. 2020). Although the use of UAVs in marine environments is limited to observations during daylight and in shallow waters or close to the surface, UAVs have provided a completely new perspective from above with longer and more detailed behavioural observations than traditional land‐ or boat‐based methods (Torres et al. 2018; Fettermann et al. 2022). In addition, as the underwater noise effect from UAVs on marine mammals is small even close to the surface, the technique provides undisturbed records of animals and behaviours (Christiansen et al. 2016). This has allowed unbiased observations of, for example, behavioural development in cetacean calves (e.g., Nielsen et al. 2019; Hamel, Torres Ortiz, and Wahlberg in press), sexual behaviours in a range of cetacean species (Ramos et al. 2023; Webber et al. 2023) and effects of whale‐watching activities on endangered species (Sprogis et al. 2023). To date, very few UAV studies on harbour porpoises have been published (but see for example Torres Ortiz et al. 2021; Brennecke et al. 2022; Hao et al. 2024). The technique, however, holds great potential for research into previously unstudied areas of harbour porpoise ecology, as shown by Torres Ortiz et al. (2021) where UAVs were used to investigate direct behavioural interactions between individual harbour porpoises during simultaneous foraging on schooling fish, and Hamel, Torres Ortiz, and Wahlberg (in press) who used UAVs to describe mother–calf interactions and maternal care.

Here, we use a large data set of UAV recordings of wild harbour porpoises from Danish waters to provide novel insights into their behavioural repertoire. We identify and describe previously unstudied foraging behaviours and provide indications for behavioural flexibility and context‐dependent foraging techniques in harbour porpoises. We also add support for UAVs as promising systems for continued studies of intra‐ and interspecific interactions of marine mammals in the field, for example, by direct observations of predator–prey and mother–calf interactions.

## 2 Materials and Methods

### 2.1 Data Collection

Visual recordings of harbour porpoises were collected ad libitum in coastal waters around the island of Funen, Denmark, from March to November 2015–2023 using quadcopter UAVs from the DJI Phantom and Mavic Series (models DJI 3, DJI 4 Pro 2.0 and Mavic III Classic, SZ DJI Technology Co. Ltd., Shenzhen, China). Data were collected only in weather conditions permitting UAV flights (wind < 12 m/s and no rain) and with wave heights less than ~0.3 m to allow detection of porpoises below the water surface. The UAV was operated by an experienced UAV pilot from shore or boat. Porpoises detected by the operator were typically followed at 10–25 m altitude for as long as possible (battery flight time 20–40 min), with the UAV real‐time video output recorded (resolution up to 4 K DCI, 4096 pixels × 2160 pixels, 60 frames/s). Porpoises were recorded both in shallow and deep waters, distinguished by the bottom being visible or not in the UAV recording. The shallow areas typically had sandy bottoms, with a varying presence of stones, macroalgae and seagrass meadows.

### 2.2 Video Analysis

All recorded videos were manually examined and studied by one person (the main author) in a video software (QuickTime Player v. 10.5) allowing the frame rate to be slowed down for detailed analysis of behaviours. Only videos with observations of porpoise during > 20 s were selected for further analysis to allow enough observation time for correct classification of behaviour. Following Torres Ortiz et al. (2021), potential foraging behaviour was identified using behavioural state classes indicative of foraging (head scan/rapid acceleration/fast turn/dive, leap or burst/bottom investigation/chasing visible prey/chasing prey not seen/prey capture). The list of potential foraging sequences was then filtered in accordance with Torres Ortiz et al. (2021) so that only sequences fulfilling criteria for being highly indicative of foraging (based on number and type of recorded behavioural states) were included as foraging sequences in the subsequent analysis. All other videos with observations of porpoise during > 20 s but without observed foraging behaviours were considered nonforaging videos. All foraging videos were then manually coded and the observed behaviour(s) categorised into a number of nonexclusive foraging modes (subbehaviours) (Table 1). The modes were constructed based on a combination of porpoise behaviour and ecological context. Detailed data (including date, location, max no. of observed individual porpoises, no. of calves, seabird presence, depth category (shallow/deep/shallow + deep, shallow: 0–5 m, deep: > 5 m, determined by bottom being generally visible in videos in shallow but not in deep areas), prey visibility (prey seen/prey not seen, prey not seen was assumed to be single prey as the density of fish schools increase their visibility) and prey type (single/school)) for identified videos (hereinafter referred to as foraging videos) was extracted into a data sheet in Microsoft Excel version 16.89 (Microsoft Corporation 2018).

**TABLE 1:** Harbour porpoise foraging modes, including the behavioural and contextual cues used for classification.

Foraging mode	Behavioural cues	Contextual cues	
		Prey	Bottom
Bottom search	Vertical position with nose down and fluke up, white belly frequently visible in UAV recording as the porpoise vertically rotate its body	Visible/not seen Single fish	Visible
Catch	Captured fish clearly visible in mouth of porpoise	Visible Single fish	Visible/not seen
Chase	Rapid acceleration, fast turn, dive, leaps or bursts (Torres Ortiz et al. 2021)	Visible/not seen Single fish/school	Visible/not seen
Cruise search	Calm and steady speed, frequent head scan (Torres Ortiz et al. 2021)	Not seen	Visible/not seen
Herd	Swimming in close proximity to school of fish, porpoise(s) Changing the direction of the school or driving it forward	Visible School	Visible/not seen
Turn	Various types of fast turns (> 180°, e.g., *side‐turn* and *flip‐turn*) at the surface	Visible/not seen Single fish	Visible/not seen
Split	Swimming through school of fish splitting it in two	Visible School	Visible/not seen

### 2.3 Statistical Analysis

Using the Excel sheet with extracted raw data, the proportion of foraging videos in relation to nonforaging videos was calculated. Similarly, descriptive statistics regarding prey visibility and distribution of foraging videos between depth categories were calculated and visualised as relative frequencies in R version 4.3.3 (R Core Team 2024) using the package ‘ggplot2’ (Wickham 2016).

Sociograms with weighted edges were constructed in R using the package ‘qgraph’ (Epskamp et al. 2012) to investigate and illustrate the association between foraging modes observed in all water depths combined, as well as in shallow and deep water respectively. No sociogram was constructed for the depth category shallow + deep as the extracted data did not allow pairing of individual identified foraging modes with a particular depth category. In addition, the foraging mode *bottom search* was excluded from the sociogram for deep water as it was impossible to determine the occurrence of this behaviour from UAV recordings. Sociograms were constructed from matrices of association between all dyads of identified foraging modes using the simple ratio index (SRI; Ginsberg and Young 1992). The SRI is the probability of two foraging modes co‐occurring in a foraging sequence, given that at least one has been observed:

$$\mathrm{SRI}=\frac{x}{x+y_{\mathrm{AB}}+y_{A}+y_{B}}$$

where *x* is the number of sequences in which foraging modes *A* and *B* were observed together, *y*_*AB*_ is the number of times *A* and *B* were observed but not together, *y*_*A*_ is the number of times only *A* was observed and *y*_*B*_ is the number of times only *B* was observed. The index ranges from 0 (*A* and *B* never observed together) to 1 (always observed together). Pairs of foraging modes with high probability of co‐occurrence were identified by calculating mean association indices. Dyads with indices at least twice the average, that is, pairs of foraging modes associated twice as frequently as what would be expected if randomly picking a pair from the matrix, were considered highly co‐occurring (Durrell et al. 2004; Gero et al. 2005; Whitehead 2008).

### 2.4 Descriptions of Specific Behaviours

From the observed behaviours in collected videos and information from constructed sociograms, a number of general foraging techniques could be identified and described based on the number of porpoises, prey type and environmental setting. As such, the techniques are general descriptions of longer foraging behaviours, sometimes including several of the more distinct foraging modes. In addition, observations of calves engaging in foraging‐like behaviours and hunting attempts were also described.

## 3 Results

### 3.1 Observations of Foraging

**FIGURE 1:**
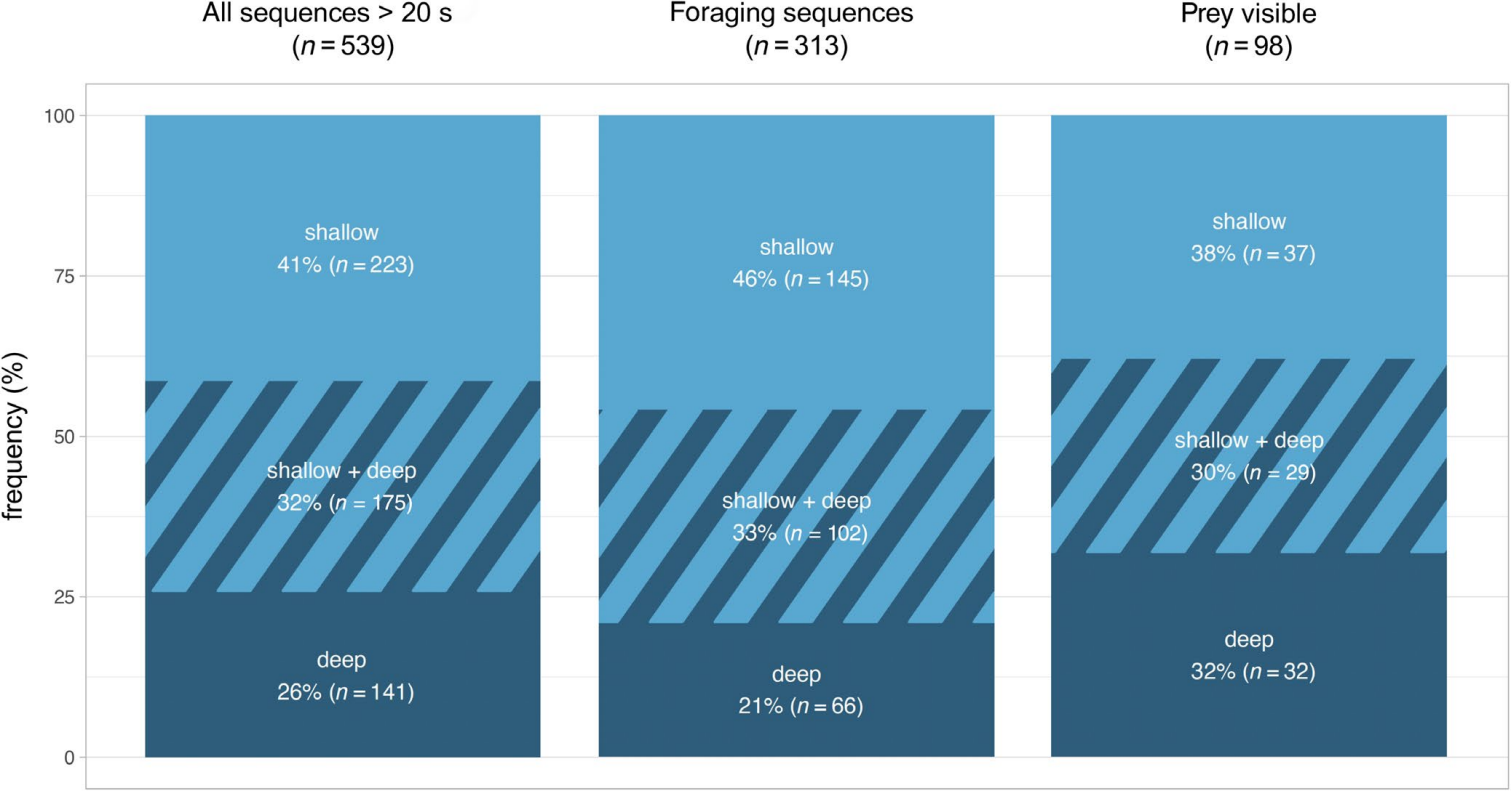
Frequency of UAV sequences from shallow, shallow + deep and deep waters. Bars represent (i) all sequences with porpoises visible longer than 20 s (‘all sequences > 20 s’), (ii) all sequences with identified foraging (‘foraging sequences’) and (iii) all sequences with active interactions with prey visible (‘prey visible’).

A total of 539 videos with porpoises visible for > 20 s (range 20 s—28 min) were recorded during 2015–2023 (Table 2), corresponding to more than 130 h of observational data. A majority of collected data (41%, *n* = 223; Figure 1) were recorded in shallow waters (depth 1–5 m) where porpoises were visible throughout the water column. In 26% (*n* = 140) of the observations, data were collected in deep waters, where the animals could not always be followed when diving (Figure 1). The remaining 32% (*n* = 176) of the data were from observations of porpoises moving between shallow and deep waters in the same video (Figure 1).

**TABLE 2:** Yearly and monthly distribution of UAV recordings of harbour porpoises used in this study, including the proportions of sequences with identified foraging behaviour.

Year	Months	No. of sequences (> 20s) without foraging	No. of sequences (> 20s) with foraging	Total	Proportion foraging
2015	Sep–Oct	3	7	10	0.70
2016	May–Sep	9	25	34	0.74
2017	Jun–Jul	3	5	8	0.63
2018	Apr–Nov	39	56	95	0.59
2019	Mar–Jul	55	100	155	0.65
2020	Apr–Sep	29	29	58	0.50
2021	Mar–Nov	14	21	35	0.60
2022	Mar–Sep	49	35	84	0.42
2023	May–Sep	25	35	60	0.58
Sum	Mar–Nov	226	313	539	0.58

**FIGURE 2:**
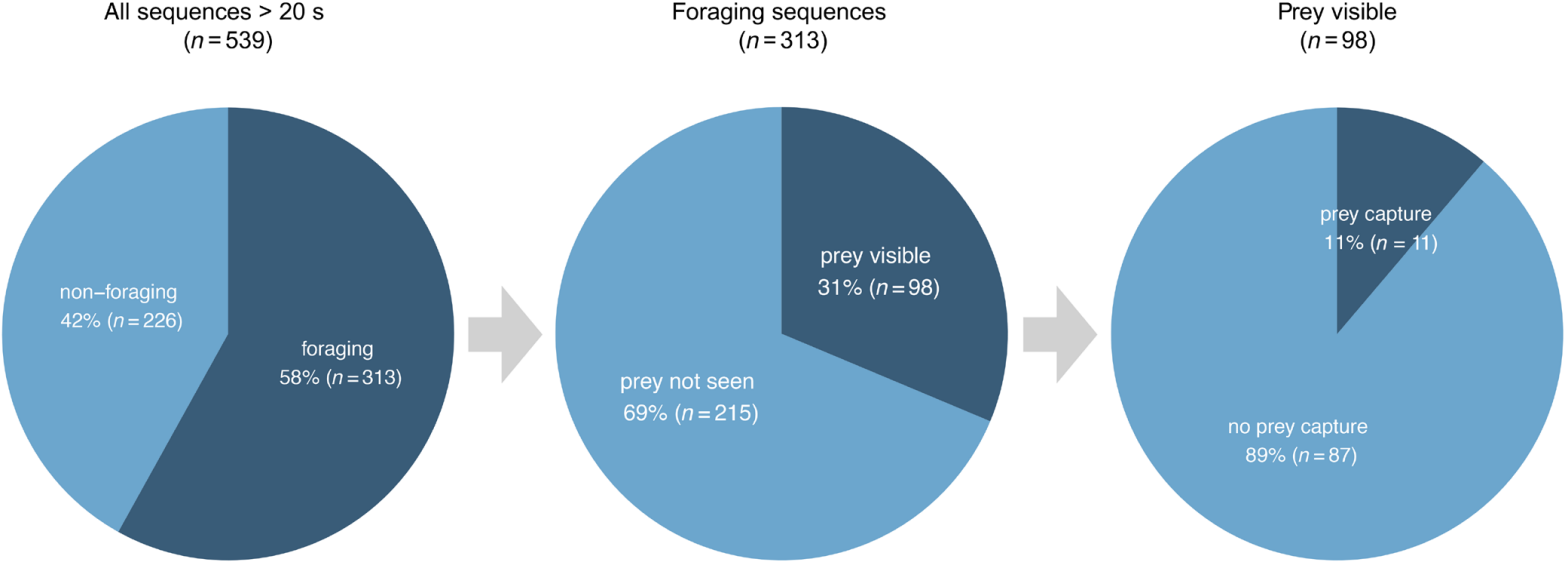
Relative distribution of UAV sequences showing (i) proportion of sequences with foraging versus nonforaging for all sequences with porpoises visible longer than 20 s (‘all sequences > 20 s’), (ii) proportion of sequences with active interactions with prey visible versus prey not seen for all sequences with identified foraging (‘foraging sequences’) and (iii) proportion of sequences with prey capture versus no prey capture for all sequences with active interactions with prey visible (‘prey visible’). Prey not seen is assumed to be single prey as the density of fish schools increases their visibility.

Foraging was identified in 58% (range 42%–74%, *n* = 313) of videos (Table 2, Figure 2). Almost half of the foraging sequences were recorded in shallow water (46%, *n* = 145). One‐third (33%, *n* = 103) were recorded in a combination of shallow and deep water, and 21% (*n* = 65) in exclusively deep water (Figure 1). While in shallow water, porpoises foraged in 65% (*n* = 145) of the videos (Table 3). In deep water, foraging was observed in 46% (*n* = 65) of the videos. When in a combination of shallow and deep water, foraging was observed in 58% (*n* = 103) of the videos.

**TABLE 3:** Depth category distribution of UAV recordings of harbour porpoises used in this study, including the proportions of sequences with identified foraging behaviour.

Depth category	No. of sequences (> 20s) without foraging	No. of sequences (> 20s) with foraging	Total	Proportion foraging
Shallow	78	145	223	0.65
Deep	75	66	141	0.47
Shallow + deep	73	102	175	0.58
Sum	226	313	539	0.58

**FIGURE 3:**
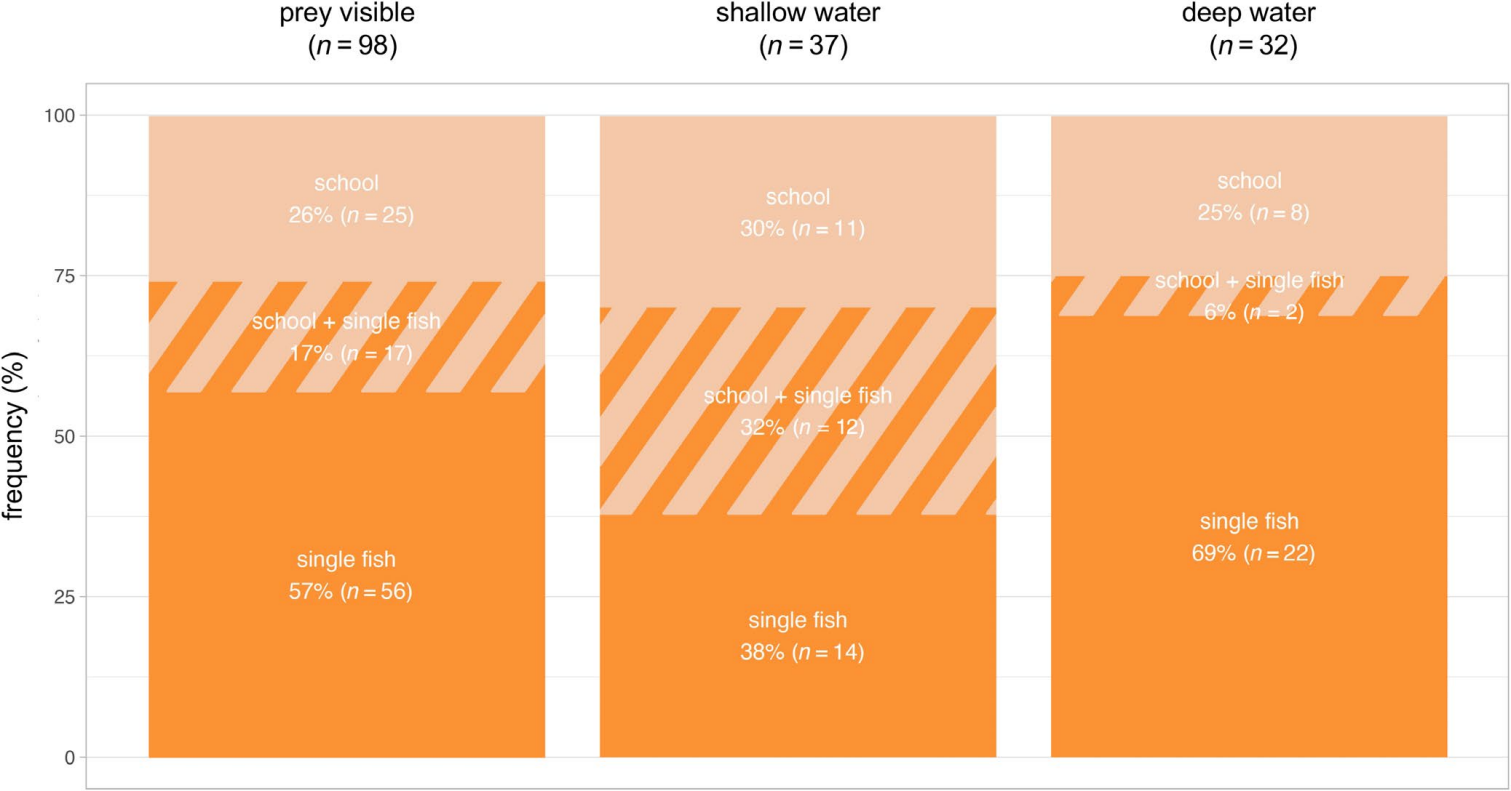
Frequency of UAV sequences from the three prey categories (school, school + single fish and single fish). Bars represent (i) all sequences with active interactions with prey visible (‘prey visible’), (ii) all sequences with active interactions with prey visible in shallow water and (iii) all sequences with active interactions with prey visible in deep water. Prey not seen is assumed to be single prey as the density of fish schools increases their visibility.

Porpoises were observed interacting with prey in 31% (*n* = 98) of foraging videos (Figure 2). In 43% (*n* = 42) of these videos, the prey was a school of fish, while in 74% (*n* = 73) of the videos, the prey was a single fish. Sometimes schooling fish and single fish were observed in the same foraging video (Figure 3). Prey were similarly observed across all depth categories: shallow 38% (*n* = 37), deep 32% (*n* = 32) and shallow + deep 30% (*n* = 29). Predation on single and schooling fish was more frequently observed in shallow and deep waters, respectively (Figure 3).

**FIGURE 4:**
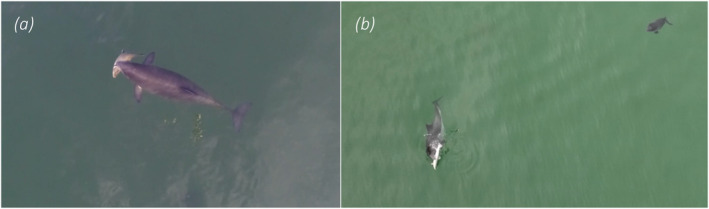
Successful prey capture of a salmonid fish, likely *Salmo trutta* , by (a) a single adult harbour porpoise and (b) a female accompanied by her calf.

Successful prey capture (with a fish clearly visible in the mouth of a harbour porpoise; Figure 4) was recorded in 11 of the 98 videos (11%) (Figure 2). Observed prey include garfish ( *Belone belone* ) and varied‐size salmonids, likely *Salmo trutta* (Figure 4), as well as probable sandeels (*Ammodytes* spp.) and clupeids (e.g., *Sprattus sprattus* and *Clupea harengus* ) (species determined from UAV images by A. Persson, personal communication).

### 3.2 Foraging Mode Associations

**FIGURE 5:**
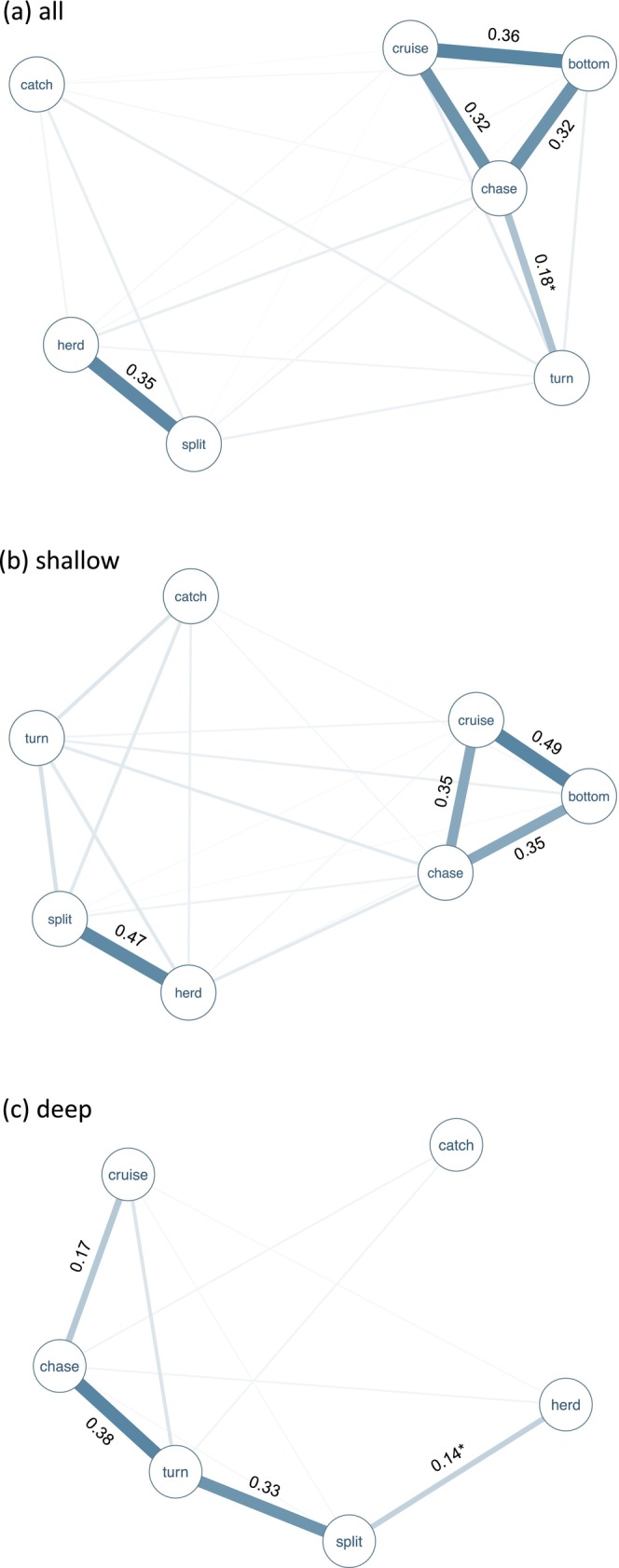
Associative networks of harbour porpoise foraging modes in (a) all water depths, (b) shallow water and (c) deep water. Associative strength between connected nodes (i.e., foraging modes) is represented by edge (line) thickness where a thicker line indicates a stronger association. Simple ratio association indices (SRI) are given for dyads with values higher than the mean, with values lower than twice the mean indicated by *.

The association matrix and sociogram on the complete dataset demonstrated presence of two distinct behavioural subnetworks (Table S1, Figure 5a). The three foraging modes *cruise search*, *bottom search* and *chase* were highly co‐occurring, with *chase* also frequently associated separately with *turn*. *Herd* and *split* were frequently expressed together and formed a second distinct subnetwork with weak associations to any of the other foraging modes. Association networks differed between shallow and deep waters (Table S1, Figure 5b,c), possibly representing a contextual effect of depth on expressed foraging behaviour. The sociogram for shallow water foraging (Figure 5b) has a similar network structure as the sociogram for all water depths, with *cruise search*, *bottom search* and *chase* being highly co‐occurring and separated from *split* and *herd* which are also strongly associated. In deep waters, *turn* is strongly associated with both *chase* and *split*, but these are almost never co‐occurring (Figure 5c). Instead, *chase* is strongly associated with *cruise search*, whereas *split* co‐occurs with *herd*.

### 3.3 Foraging Techniques

**FIGURE 6:**
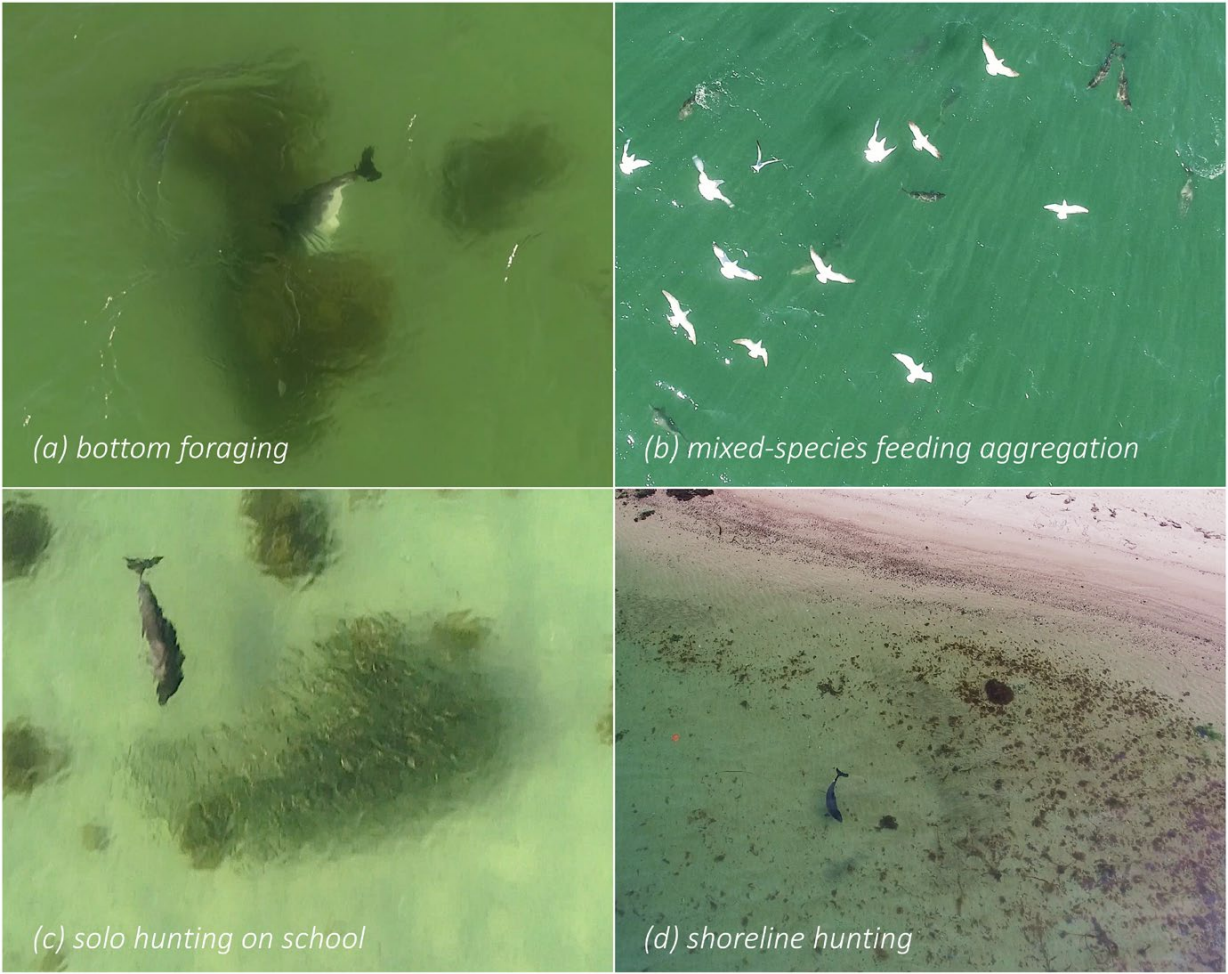
Harbor porpoises display variety of foraging techniques and hunting behaviours, including (a) *bottom foraging*; vertical forage in the sand or by rocks and algae at the sea bed with head‐down and fluke‐up, (b) *mixed‐species feeding aggregation*; a special case of group hunting on schools of fish where multiple porpoises and seabirds predate on the same prey resource, (c) *solo hunting on school*; interaction by a single HP with school of fish, for example, affecting school movements and (d) *shoreline hunting*; a special case of group/solo hunting with interaction by single or multiple HPs with school of fish in very close proximity to the shoreline (seemingly using the shoreline as a barrier). Note that longer sections of the behaviours are available as supplemental video through Dryad: https://doi.org/10.5061/dryad.dv41ns26k.

**FIGURE 7:**
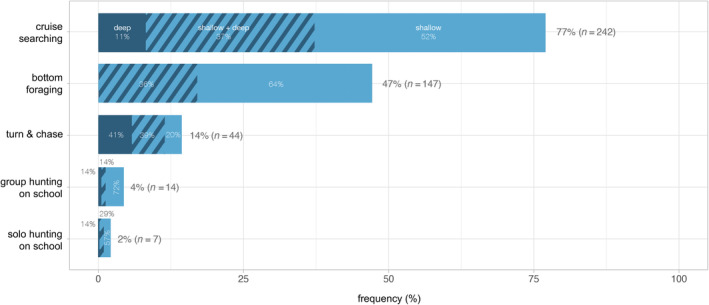
Frequency of harbour porpoise foraging techniques identified in this study. Bars show relative proportions split up between depth categories (shallow, shallow + deep and deep) for each technique. Note that the foraging techniques are nonexclusive and can co‐occur in the same sequence, hence the sum of all bars exceeds 100%.

**FIGURE 8:**
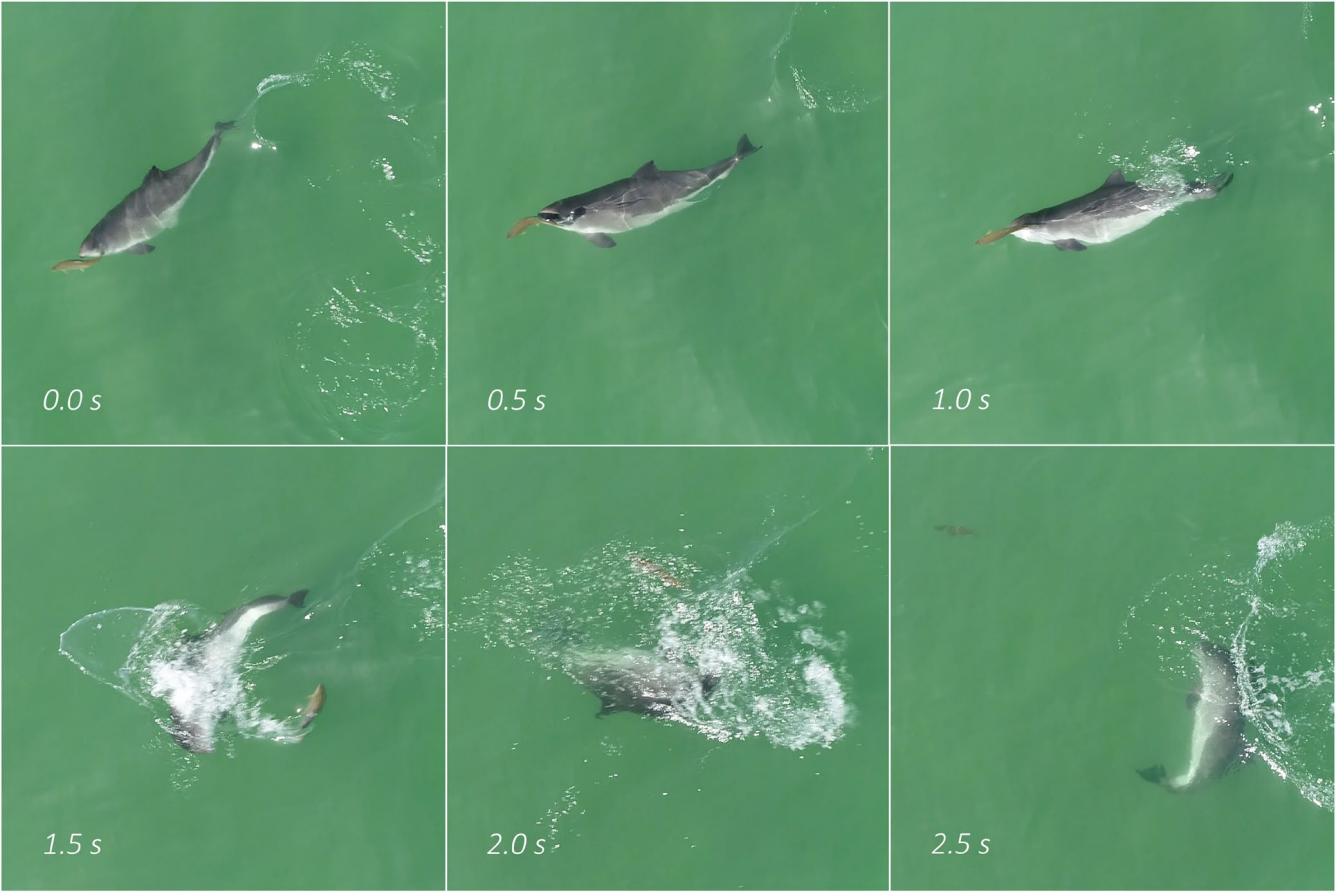
Photo sequence from video recording of the *turn & chase* foraging technique. Time between subsequent photos is 0.5 s. Note that a longer section of the behaviour is available as supplemental video through Dryad: https://doi.org/10.5061/dryad.dv41ns26k.

Based on observed behaviours and constructed sociograms, in combination with number of porpoises, prey type and environmental setting, six foraging techniques could be identified and described (Table 4). Representative examples of all identified foraging techniques are available as supplementary video material through Dryad: https://doi.org/10.5061/dryad.dv41ns26k. *Bottom foraging* (Figure 6a) could only be observed in shallow water but nevertheless represents one of the most frequently observed foraging techniques exhibited by both single individuals and aggregations of porpoises, with the associated foraging mode *bottom search* observed in close to half (*n* = 147) of all foraging sequences (Figure 7). *Cruise searching* was seen in 77% (*n* = 242) of all sequences (Figure 7), making it the most frequently observed technique. *Group hunting on school* includes collaborative group hunting as described by Torres Ortiz et al. (2021) and events with multiple porpoises and seabirds predating on the same school (referred to as a mixed‐species feeding aggregation; Figure 6b). The group sizes observed during this type of foraging ranged from 2 to 12 individuals (confirmed separate individuals). These group hunting behaviours were observed in only 14 (4%) of the sequences (Figure 7), with confirmed collaboration occurring in two of these (15%) (the same sequences analysed in Torres Ortiz et al. 2021). Groups of two or more porpoises were never observed actively chasing or directing the movements of the same single prey fish, instead situations with more than one porpoise and a single prey indicate possible observational learning (see section *Foraging behaviour in calves* below). *Solo hunting on school* (single individuals interacting with schools of fish; Figure 6c) was observed in seven sequences (2%; Figure 7). *Shoreline hunting* (Figure 6d) was observed in six sequences (2%; Figure 7) and was identified as a special case of *group/solo hunting on school* in which porpoises seemingly use the shoreline as a barrier to control school movements. Lastly, *turn & chase* (Figure 8) (active chase of single prey close to the surface with various types of fast turns, rapid accelerations and fast halts) was observed in 44 (14%) of the sequences (Figure 7). At least two different types of turns could be differentiated in our material. The first is a *side‐turn* in which the porpoise rolls 90° so that its flank (rather than the dorsal fin) lies closest to the surface before it changes heading abruptly. The second type is a *flip‐turn* in which the porpoise abruptly reverses direction along its vertical axis by curling its rostrum towards its flukes (similar to the turn executed by a competitive swimmer when reversing direction at the end of a swimming pool). Some sequences with classified foraging contained more than one of the identified foraging techniques, while others could not be attributed to any of the techniques.

**TABLE 4:** Descriptions of harbour porpoise (HP) foraging techniques. #HP depicts the total number of individual porpoises (range, min–max) observed in recordings where the behaviour was observed, and does not necessarily represent the number of individuals participating in the technique.

Foraging technique	#HP (min–max)	Observed prey type/species	Observed environmental context	Description	Note
Solo hunting at school	1	School chase/catch of small schooling fish	Shallow/deep water	Interaction by single HP with school of fish; affecting school movements, splitting school and sometimes achieving separation of individual prey allowing succeeding active chase/capture.	—
Turn & chase	1–4	Single chase/catch of salmonids (e.g. *Salmo trutta* )	Shallow/deep water	Active chase of prey close to the surface with various types of fast turns (180°) (e.g., *side‐turn* and *flip‐turn*), rapid accelerations and fast halts. Predominantly performed by a single HP, with a second HP (e.g., calf) occasionally participating less actively.	—
Shoreline hunting	1–5	School chase/catch of small schooling fish (probable *Ammodytes* spp.)	Beach shoreline	Interaction by single or multiple HPs with school of fish in very close proximity to the shoreline (seemingly using the shoreline as a barrier); affecting school movements, splitting school and sometimes achieving separation of individual prey allowing succeeding active chase/capture.	Special case of group/solo hunting on school
Bottom foraging	1–8	Single fish chase/catch of small fish	Shallow water	Single or multiple HPs swimming close to the seabed, head scanning, investigating the bottom (sandy and/or patchy with stones/algae) and repeatedly stopping to vertically forage with head‐down and fluke‐up.	UAV data limited to observations in shallow water. Includes bottom grubbing as described by Lockyer et al. (2001).
Cruise searching	1–8	Single fish/school	Shallow/deep water	Single or multiple HPs swimming with calm and steady speed close to surface while frequently scanning with head, occasionally sudden increase in speed towards detected prey at, e.g., seabed and followed by *bottom foraging*.	—
Group hunting on school	2–12	School chase/catch of small schooling fish	Shallow/deep water	Interaction by multiple HPs with school of fish; affecting school movements, splitting school and sometimes achieving separation of individual prey allowing succeeding active chase/capture.	Includes collaborative group hunting as described by Torres Ortiz et al. (2021), and events with multiple porpoises and seabirds predating on the same school (referred to as a mixed‐species feeding aggregation).

### 3.4 Foraging Behaviour in Calves

**FIGURE 9:**
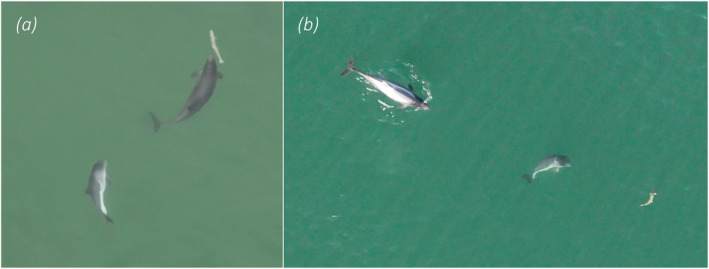
Potential observational learning by a harbour porpoise calf in presence of its mother, with (a) the calf closely following the hunt of a salmonid fish while fixating its head direction on the prey, and (b) the mother increasing her distance to the prey and the calf mimicking hunting behaviours previously performed by the mother.

Calves less than 1 year old were clearly discernible in the UAV videos, based on time of year of observations (April–Nov, and given that June–July is peak calving season (Sørensen and Kinze 1994)) and their small size relative to mother (presuming the associated adult as the mother) (Stepien et al. 2023). Calves were observed in 120 (38%) of the foraging sequences, with a range of 1–4 calves in every video, and a calf‐to‐adult ratio of 0.24. Young calves were often observed ‘waiting’ alone close to the surface, or seemingly passively following along their mother and other adult individuals that actively engage in foraging. On some occasions, calves are seen actively investigating seaweed and jellyfish on the surface and performing behaviours resembling the *turn & chase* technique. Our data also include some observations of mother–calf pairs engaged in hunting on the same prey target, typically a large salmonid hunted by the mother while the calf followed with its head direction fixed on the prey. The mother was sometimes seen increasing her distance from the prey, seemingly to allow the calf to perform behaviours mimicking the mother's (Figure 9).

## 4 Discussion

### 4.1 Daytime Foraging Behaviour

Prior studies on tagged porpoises in Danish waters suggest that porpoises are required to forage > 60% of the time to maintain their high‐field metabolic rate (Rojano‐Donate et al. 2024). It is therefore not unexpected that almost 60% of the sequences in our extensive UAV data set include visually detectable foraging behaviours (Table 2). Data collected by UAV are, however, limited to observations during daylight hours, and at water depths and weather conditions where animals remain visible. Single porpoises with less active movements could to a minor degree also be underrepresented in our data due to detectability bias. Even so, Danish porpoises spend the majority of their time (68%) at 0–5 m depth (Teilmann, Larsen, and Desportes 2007), supporting our contention that UAV observations can capture a large proportion of their daytime behaviours. Dive profiles from tagged harbour porpoises suggest that they switch between near‐surface, pelagic and benthic foraging during daytime to mostly pelagic foraging during night (Wisniewska et al. 2016; Rojano‐Donate et al. 2024), indicating a behavioural flexibility in response to changing foraging conditions possibly driven by light availability.

Given the methodological restrictions mentioned above, our UAV data only allow us to speculate on potential differences in foraging behaviour between shallow and deep waters. The difference in observed foraging frequency between shallow (65%) and deep (47%) areas (Table 3) could reflect methodological bias, although it is also possible that it represents a true behavioural difference between habitats with a higher overall proportion of foraging in water depths < 5 m. This would then be similar to other coastal species of odontocetes, such as bottlenose dolphins ( *Tursiops truncatus* ), which show preferences for foraging at shallow depths (Torres and Read 2009; Weiss 2006). Similarly, the difference in observed prey types between shallow and deep water (Figure 3) is likely to reflect a combination of true differences in prey availability and detectability bias, with most schools of fish in deep water remaining undetected.

The associations between foraging modes provide support for behavioural differences between depth contexts, with network structures differing between shallow and deep waters (Figure 2b,c). In shallow water, *cruise search*, *bottom search* and *chase* are highly associated, thus the foraging techniques *cruise searching* and *bottom foraging* seem to represent common foraging techniques which are often performed in combination in shallow habitats. This is supported by observational notes made during review of collected sequences that porpoises are repeatedly seen *cruise searching* in shallow water for a period before setting off towards the bottom which is often followed by *bottom foraging* and sometimes also visible catch of prey. These two behaviours are also often seen performed in recurring sequences, with porpoises returning to *cruise searching* after a period of *bottom foraging*.

Two other behaviours, *herd* and *split* often co‐occurred in shallow waters. This behavioural combination was observed every time schools of fish were hunted, thus representing *group/solo hunting on school*. Our observations suggest that porpoises repeatedly herd and split the school into smaller and smaller units to eventually achieve so‐called *flash expansion* where individual prey disperse in different directions (Pitcher and Wyche 1983). The porpoise then chases individual fish which are no longer protected in the school. During group hunting, porpoises sometimes collaborate to achieve splitting of fish schools by some individuals moving alongside the school seemingly directing school movements while others cross through the school (Torres Ortiz et al. 2021). Similar manipulation of fish schools into smaller units has also been reported during mixed‐species feeding aggregations of common dolphins ( *Delphinus delphis* ), bottlenose dolphins ( *Tursiops truncatus* ), spotted dolphins ( *Stenella frontalis* ) and striped dolphins ( *S. coeruleoalba* ) (Clua and Grosvalet 2001), as well as by killer whales ( *Orcinus orca* ) (Vabø and Nøttestad 2003). Although repeated attacks on fish schools increase prey capture success (Thiebault et al. 2016), the repeated herding and splitting of a school by harbour porpoises to achieve separation of individual prey requires further study to investigate if it represents a novel porpoise foraging behaviour.

We propose that porpoises sometimes use the shoreline as a barrier to achieve control over school movements to make it split (Figure 3d). This could be a technique used by porpoises to allow more successful hunts on schools of fish in absence of other porpoises to collaborate with or to more efficiently catch prey. Similar use of natural barriers during hunting has been described for bottlenose dolphins (Sargeant et al. 2005; Torres and Read 2009; Pace and Pedrazzi 2024) and Guiana dolphins ( *Sotalia guianensis* ) (Pierry et al. 2023), but not for porpoises. Detailed studies investigating whether the use of the shoreline increases hunting efficiency for porpoises, for example, decreases time spent handling school and/or number of times splitting the school before flash expansion is achieved, could provide further insights into the mechanisms behind this foraging technique and its prevalence in different genera of odontocetes.

The fact that foraging modes *turn* and *chase* are highly associated only in deep waters suggests that the related technique *turn & chase* is dependent on not only depth but also the habitat which the prey associates with. We have almost exclusively observed *turn & chase* during hunt of large salmonid prey (likely *Salmo trutta* ). *Turn & chase* has been observed in several areas and by at least two different porpoises, although most observations (75%) are from a female porpoise recognisable by scars and marks on her body. This female has been observed utilising this foraging technique during 5 of the 7 years in which she has been resighted in the same area close to Kerteminde Harbour, Denmark. The female is often seen foraging on large salmonids in close presence of its calf, which is following closely and keeping its head focused on the mother and the prey fish.

The reason for porpoises performing various types of turns during foraging is not clear from data presented here. It could represent locomotor strategies by porpoises to compensate for their nonequal manoeuvrability in relation to smaller and more agile prey (Maresh et al. 2006). In particular, the motivation for a porpoise to perform a *side‐turn* would be interesting to investigate further. This type of turn is probably more challenging for the porpoise to perform than the flip‐turn as the porpoise's body is less flexible laterally along its spine than when curling its rostrum towards its flukes. Perhaps the side‐turn is a way to adjust the biosonar signal to prevent back‐scatter, or some other acoustic phenomenon that makes it hard to pinpoint a rapidly moving object when targeting a single fish close to the surface (Wei et al. 2023).

### 4.2 Possible Learning Through Social Information Transfer

Our interpretation of the behaviours in mothers and calves described above is that the calf is actively observing the hunt and sometimes also ‘practicing’, by mimicking the movements previously performed by the mother when she moves away from the prey and ‘gives way’ for the calf to have a try. Given that calves at a young age perform behaviours closely resembling the foraging behaviours expressed by adults and possibly actively observe hunting attempts, we propose that harbour porpoises acquire at least some of their foraging techniques through social learning. Our suggestion is further corroborated by mother–calf observations made by Hamel, Torres Ortiz, and Wahlberg (in press). Learning is fundamental for some odontocete foraging behaviours (Sargeant and Mann 2009; Baird 2000; Guinet and Bouvier 1995; Weiss 2006) and mothers have a key role in transmission of behavioural strategies to their calves (Bender, Herzing, and Bjorklund 2009; Sargeant et al. 2005).

The idea that porpoises are capable of social learning through observation was proposed 50 years ago (Amundin and Amundin 1971) but has since received little attention. This is probably partly due to the methodological limitations restraining all observational studies of porpoise behaviour, but might also be influenced by the often‐stated view that porpoises are nonsocial animals (even though it has been noted that this view may be more caused by limitations in observation methods than the true nature of the species; see Read 1999). Social learning is, however, not necessarily restricted to grouping species, as also nongrouping species are regularly exposed to social stimuli from conspecifics (Webster, Laland, and Skelhorn 2017). For a predominantly solitary or nonsocial animal, such exposure might take place during temporary aggregation with others during exploitation of a patchy resource (e.g., fish school) or during particular stages of their life (e.g., during reproduction and nursing).

Regardless of the social structure of harbour porpoises, social information is highly likely to be transmitted between individuals, especially between mother and offspring during the preweaning period. Our observations of porpoise calves seemingly practising behaviours used for foraging by close adults and of mother–calf interactions during accompanied foraging support this idea. Presence of learning processes could be further investigated by exploring if harbour porpoise mothers, for example, take longer time to capture prey and/or adapt their body movements while hunting in presence of their calves (Bender, Herzing, and Bjorklund 2009). If social learning occurs in harbour porpoises, cultural transmission of hunting techniques could be possible as social learning is necessary for cultural transmission of behaviour (Van Schaik 2010).

### 4.3 Foraging Rates and Metabolic Needs

Harbor porpoises tagged with high‐resolution tags (DTAGs) in Danish waters had extremely high foraging rates. They were observed to forage nearly continuously during the 6–43 h they were monitored (Wisniewska et al. 2016; Rojano‐Donate et al. 2024). These porpoises targeted up to 200 and 500 small fish or shrimp every hour during day and night time, with an estimated capture success rate of > 90%. UAV recordings are unlikely to accurately capture most such small prey pursuits, especially as tagged harbour porpoises show elevated engagement in foraging dives and prey capture attempts during nighttime (Rojano‐Donate et al. 2024) when UAV data are lacking. Even so, there is little evidence in our large visual dataset supporting such ultrahigh foraging rates. Instead, we observe large variations in foraging behaviour and porpoises spending several minutes chasing the same individual fish. We propose that this discrepancy reflects true behavioural variations in harbour porpoises. Following release, tagged animals might forage intensely on small prey to compensate for lost hunting opportunities during entrapment and therefore might be unlikely to express their complete range of foraging behaviours (Hoekendijk et al. 2017; Wisniewska et al. 2017). On the other hand, UAV data are unable to describe foraging at depth and night when tagged animals foraged the most (Rojano‐Donate et al. 2024). Combining results from tagged animals and UAV observations present harbour porpoises as opportunistic predators with context‐dependent behavioural flexibility, allowing these small odontocetes to sustain their high metabolism and make use of the prey resources currently available.

Harbor porpoises consume up to 10% of their body weight each day (Kastelein, Hardeman, and Boer 1997; Lockyer et al. 2003), with an average daily energy requirement for an adult porpoise of 17–20 MJ (Andreasen et al. 2017; Rojano‐Doñate et al. 2018). To meet their predicted metabolic demands, an alternative to eating hundreds of small prey is to target larger and/or more energy‐dense prey. The *turn & chase* technique is often seen performed for several minutes at a time, often targeting a large salmonid. Assuming a *Salmo trutta* with a weight of 1.5 kg and an energy density of 5611 J/g wet weight (Pizzul et al. 2009) reveals that one such large (~30 cm) salmonid prey could potentially contain 8.4 MJ, which corresponds to almost half of the required daily energy intake for an adult harbour porpoise (estimated to 17–20 MJ by Rojano‐Doñate et al. 2018). In this respect, it is interesting to note that we mostly see this behaviour performed by pregnant and lactating females which have especially high energy demands (Lockyer and Kinze 2003; Gallagher, Stern, and Hines 2018) motivating investment of considerable time and energy into hunt on energy‐rich prey. Visual observations of lactating female porpoises hunting and catching large salmonids have also been made on the US West Coast (Elliser et al. 2020). The behaviours reported by Elliser et al. (2020) are very similar to the behaviours that we have observed in Danish waters, with porpoises tightly turning and circulating at the surface while chasing after prey (here termed *turn & chase*), and then carrying the fish sideways in the mouth after catch (Figure 4). Although it is perhaps not surprising, it is still interesting to have the same specific foraging behaviour documented for two separate porpoise populations on either side of the world.

### 4.4 Conflicts With Human Activities

Given their high metabolic activity, harbour porpoises are considered especially vulnerable to disturbance influencing their foraging success. Coastal areas are often highly trafficked and porpoises might encounter vessel noise up to 89% of their time in inner Danish waters (Wisniewska et al. 2018), with potential negative impacts on foraging success and long‐term fitness consequences (Tougaard, Wright, and Madsen 2015). Our study shows that coastal areas are important foraging grounds for porpoises and provide support for concerns raised about negative effects of noise pollution on marine life in these areas. In addition, coastal areas in Denmark and elsewhere are widely used for net fishing. Bycatch in static fishing nets is the main cause of death for porpoises, with an estimated ~300 porpoises bycaught in the Belt Sea each year (Kindt‐Larsen et al. 2023). Based on our behavioural observations, we postulate that some of the observed foraging behaviours might generate a higher bycatch risk than others. Given that porpoises rely mostly on their biosonar for spatial orientation and foraging and that the echolocation signals are emitted in a highly directional and very narrow beam from their head (Koblitz et al. 2012), our impression is that porpoises are likely to occasionally be less aware of their surroundings and therefore more prone to entanglement in prospective nets. We propose possible decreased peripheral awareness during some of the observed foraging behaviours, for example, during fast acceleration, rapid turning and when moving along the bottom in a vertical position during bottom foraging. When focused on bottom exploration and active chase of prey, porpoises are likely to be less responsive to sensory cues with information from other parts of their surrounding with an increased risk of nets being undetected (Martin and Crawford 2015), discussing this subject in‐depth regarding bycatch of marine birds. Declined availability and nutritional condition of larger and schooling prey species in inner Danish waters (ICES 2019; Receveur et al. 2022) could then further increase bycatch risk if porpoises increase their effort targeting smaller benthic prey. Elucidating whether context‐dependent differences in bycatch risk exist with regard to performed foraging behaviours and targeted prey could help reduce bycatch, but need to be investigated in more detail.

## 5 Conclusions

This study provides the first record of previously undescribed foraging strategies used by harbour porpoises. Results from visual and network analyses indicate use of context‐dependent foraging strategies, with a broad collective repertoire of foraging behaviours, suggesting that porpoises are flexible predators that use conditional foraging strategies and adapt their behaviour in response to environmental characteristics. Such behavioural plasticity is not surprising for opportunistic predators like the harbour porpoise, as they inhabit a broad variety of marine habitats with diverse conditions for both predators and prey. Furthermore, our visual observations of foraging in almost 60% of recorded UAV videos in Danish coastal waters during daylight hours support results from acoustic studies stating that porpoises spend a considerable part of their time foraging. This study provides an important contribution to our understanding of harbour porpoise ecology and foraging behaviour, which is valuable information for conservation when working to reduce conflicts with human activities in coastal areas.

## Author Contributions

**Johanna Stedt:** conceptualization (lead), data curation (lead), formal analysis (lead), funding acquisition (equal), methodology (lead), resources (supporting), visualization (lead), writing – original draft (lead), writing – review and editing (lead). **Héloïse Hamel:** investigation (equal), writing – review and editing (supporting). **Sara Torres Ortiz:** investigation (equal), writing – review and editing (supporting). **Jakob Højer Kristensen:** investigation (supporting), writing – review and editing (supporting). **Magnus Wahlberg:** conceptualization (supporting), funding acquisition (equal), resources (lead), writing – review and editing (supporting).

## Conflicts of Interest

The authors declare no conflicts of interest.

## Permits

The UAVs were operated under Permit No. 5032864 from Trafik‐, Bygge‐og Boligstyrelsen (Danish transport, construction and housing authority).

## Supporting information


Table S1

## Data Availability

Datasets containing the data analysed in this paper and a selection of videos with the observed behaviours and foraging techniques described are available at the open data publishing platform Dryad, https://doi.org/10.5061/dryad.dv41ns26k.
