# Supplementary material for: Harbour Porpoises Are Flexible Predators Displaying Context‐Dependent Foraging Behaviours
Source: Ecol Evol. 2024 Dec 4;14(12):e70671. doi: 10.1002/ece3.70671 (PMC11615644; doi:10.1002/ece3.70671)
Supplement: Supplementary file 1 — Table S1 [file ECE3-14-e70671-s001.pdf]

SUPPLEMENTARY

Table S1. Simple ratio association indices (*SRI*) and number of sequences (in brackets) for dyads of harbor porpoise foraging modes observed in all water depths, as well as in shallow and deep water respectively. Dyads with association indices above the mean (all: 0.10; shallow 0.12; deep 0.08) indicated by * and dyads with association indices at least twice the mean shown in bold.

	bottom search	catch	chase	cruise search	herd	turn	split
**all (313)**							
bottom search (147)		-	-	-	-	-	-
catch (11)	0.02 (7)		-	-	-	-	-
chase (211)	**0.32 (100)***	0.02 (10)		-	-	-	-
cruise search (242)	**0.36 (134)***	0.01 (5)	**0.32 (147)***		-	-	-
herd (21)	0.02 (7)	0.03 (2)	0.05 (21)	0.02 (11)		-	-
turn (66)	0.05 (17)	0.05 (7)	0.18 (64)*	0.06 (31)	0.03 (5)		-
split (14)	0.01 (3)	0.05 (2)	0.03 (14)	0.01 (7)	**0.35 (12)***	0.04 (6)	
**shallow (145)**							
bottom search (94)		-	-	-	-	-	-
catch (5)	0.03 (5)		-	-	-	-	-
chase (90)	**0.35 (63)**	0.03 (5)		-	-	-	-
cruise search (125)	**0.49 (87)**	0.01 (3)	**0.35 (73)**		-	-	-
herd (14)	0.03 (6)	0.06 (2)	0.08 (14)	0.03 (8)		-	-
turn (14)	0.06 (10)	0.10 (3)	0.08 (14)	0.04 (10)	0.09 (4)		-
split (9)	0.02 (3)	0.09 (2)	0.05 (9)	0.02 (4)	**0.47 (9)**	0.12 (4)	
**deep (66)**							
bottom search (0)		-	-	-	-	-	-
catch (4)	NA		-	-	-	-	-
chase (53)	NA	0.03 (3)		-	-	-	-
cruise search (26)	NA	0.00 (0)	**0.17 (18)***		-	-	-
herd (3)	NA	0.00 (0)	0.03 (3)	0.02 (1)		-	-
turn (31)	NA	0.03 (2)	**0.38 (30)***	0.08 (7)	0.00 (0)		-
split (2)	NA	0.00 (0)	0.02 (2)	0.02 (1)	0.14 (1)*	**0.33 (1)***	
